# A Novel *de novo* Mutation in *EBF3* Associated With Hypotonia, Ataxia, and Delayed Development Syndrome in a Chinese Boy

**DOI:** 10.3389/fgene.2021.676832

**Published:** 2021-07-22

**Authors:** Yanru Huang, Libin Mei, Yangdan Wang, Huiming Ye, Xiaomin Ma, Jian Zhang, Meijiao Cai, Ping Li, Yunsheng Ge, Yulin Zhou

**Affiliations:** ^1^Women and Children’s Hospital, School of Medicine, Xiamen University, Xiamen, China; ^2^United Diagnostic and Research Center for Clinical Genetics, School of Public Health, Xiamen University, Xiamen, China; ^3^Xiamen Key Laboratory of Reproduction and Genetics, Xiamen, China

**Keywords:** hypotonia, ataxia, delayed development syndrome, *EBF3*, pathogenic mutation, trio whole-exome sequencing, c.589A > G

## Abstract

**Objective:**

Global developmental delay has markedly high phenotypic and genetic heterogeneity, and is a great challenge for clinical diagnosis. Hypotonia, ataxia, and delayed development syndrome (HADDS), first reported in 2017, is one type of global development delay. The aim of the present study was to investigate the genetic etiology of a Chinese boy with global developmental delay.

**Methods:**

We combined clinical and imaging phenotyping with trio whole-exome sequencing and Sanger sequencing to the patient and his clinically unaffected parents. A luciferase reporter and immunofluorescence were performed to detect the effect of mutation on transcriptional activity and subcellular localization.

**Results:**

The patient presented with several previously unreported symptoms in the patients with HADDS, including hemangiomas, mild hearing abnormalities and tracheomalacia. A novel *EBF3* c.589A > G missense mutation (p.Asn197Asp, p.N197D) was identified in the patient but not in his parents. By constructing the plasmid and transfecting HEK293T cells, *EBF3*-N197D mutant showed impaired activation of luciferase reporter expression of the p21 promoter, and the mutant affected its entry into the nucleus.

**Conclusion:**

To the best of our knowledge, this is the first report of *EBF3* pathogenic mutation which associated with HADDS in the Chinese population. Our results expand the phenotypes and pathogenic mutation spectrum of HADDS, thus potentially facilitating the clinical diagnosis and genetic counseling of HADDS patients.

## Introduction

Global developmental delay refers to a significant retardation of a child’s growth and development relative to its peers (Shevell et al., 2003). It affects cognitive or thinking skills, motor skills, social and emotional, language, and vision.^1^ Different individuals present with different levels of severity, thus deterring its clinical diagnosis. The etiology of global developmental delay includes exogenous and genetic (non-metabolic or metabolic) factors (Papavasiliou et al., 2000; Michelson et al., 2011). Advancements in modern biotechnology and genetic testing technology have facilitated the diagnosis of children with global developmental delay (Srour and Shevell, 2014). Whole-exome sequencing (WES), whole-genome sequencing (WGS), and genome-wide microarrays have facilitated the identification of several candidate genes associated with developmental delays.

Herein, we curated data from one family, which included a boy with global developmental delay, born to non-consanguineous parents with a normal phenotype. The patient presented with motor delay, mental retardation, language delay, fontanelle closure delay, hypotonia, funnel chest, cryptorchidism, hemangioma, and febrile seizures. No obvious microdeletion or microduplication was detected using genome-wide microarray analysis (Affymetrix CytoScan 750K). A novel *EBF3* gene c.589A > G missense mutation was detected in the patient via Trio-WES but not in the parents. To our knowledge, there are no more than 20 cases, who had *de novo* variants in *EBF3* and a distinct neurodevelopmental syndrome, reported worldwide since the disease first reported in 2017 (Chao et al., 2017; Harms et al., 2017; Sleven et al., 2017). Besides, this is the first report about HADDS from China.

## Materials and Methods

### Genomic DNA Extraction and Genome-Wide Copy Number Variation Analysis

Genomic DNA was extracted from peripheral blood leucocytes of the proband and his parents using the QIAamp Blood Mini Kit (QIAGEN, Hilden, Germany) following manufacturer protocol. CMA-SNP array analysis of the prband’s DNA was performed using the Affymetrix^®^ CytoScan^TM^ 750K Array (Affymetrix, Santa Clara, CA, United States) following the manufacturer’s recommended protocols. When all quality control tests were passed, we analyzed deletions of ≥ 50 kb (marker ≥ 20 kb), repeats of ≥ 100 kb (marker ≥ 20 kb), and homozygous chromosomal fragments of > 5 Mb.

### Whole-Exome Sequencing and Bioinformatics Analysis

The genomic DNA of the proband and his parents was digested using segmentase (BGI, Shenzhen, China) into 100–500-bp fragments. Thereafter, 280–320-bp-long fragments were subjected to enrichment, blunting, A-tailing, and adapter ligation, followed by PCR amplification for library preparation. The DNA library thus generated was used to capture and collect DNA from the target exons and adjacent splice sites, using the BGI V4 probe (58.7M). Finally, the MGISEQ-2000 sequencing platform (MGI, Shenzhen, China) was used for PE100 + 100 sequencing. The quality control indicator for the sequencing data was an average effective sequencing depth of ≥ 100 × for the target region, where 95% of the sites had an average depth of 20×.

After the quality control analysis of the raw data, reads were aligned with the UCSC hg19 human reference genome, using BWA to eliminate duplicates. GATK was used to calibrate SNV and INDEL base quality scores and genotype analysis.

### Validation by Sanger Sequencing

One pair primers (*EBF3*-7F: CGAAAGTCGCAGCTATTATCAT; *EBF3*-7R: TTAGACTTGATGAATCTGGCATAC) were designed using Oligo 6 to amplify the candidate regions of the mutation in *EBF3* gene (NM_001005463.2) identified by WES. Then, forward and reverse Sanger sequencing were performed using ABI PRISM 3730 gene analyzer (Applied Biosystems, California, United States).

### Cell Culture

HEK293T cell line (ATCC, Rockville, MD, United States) was cultured in DMEM medium (GIBCO, Invitrogen Corporation, NY, United States) containing penicillin (final concentration of 100 U/ml, Sigma, St. Louis, MO, United States), streptomycin (final concentration of 100 μg/ml, Sigma, St. Louis, MO, United States) and 10% fetal bovine serum (FBS, Hyclone, Logan, UT, United States).

### Expression Analysis

To characterize the effects of the *EBF3* mutation at the cellular level, transient cell transfections were performed in HEK293T cell line with WT or mutated *EBF3* mRNA expressed as fusions to the C-terminus of Flag (pcDNA-Flag-C, Invitrogen Corporation, NY, United States). Mutation was introduced via site-directed mutagenesis (QuickMutation^TM^ Site-Directed Mutagenesis Kit, Beytime, ShangHai, China) with the forward primer 5’-CTAGGATCCATTACAACACAGTCAGCACT-3’ and the reverse primer 5’-ATTGAATTCTCTTCTGTTTCATGCCGTAG-3’. All of the inserts were systematically verified by sequencing. The transfection was performed by incubating 2 μg of fusion protein construct using Lipofectamine 3,000 (Thermo Fisher Scientific, Pittsburgh, PA, United States) according to the manufacturer’s instructions.

Firefly Luciferase Reporter Gene Assay Kit (Beytime, ShangHai, China) was used to detect the transcriptional activity of *EBF3*-WT and *EBF3*-N197D.

Immunofluorescence was used to detect the subcellular localization and captured by Laser Scanning Confocal Microscopy (Zeiss LSM, Zeiss, Germany).

The scraped cells were transferred to a 1.5 mL precooled microcentrifuge tube with 1 mL precooled PBS. Centrifuge at 4°C, collect cells at 1,000 g for 3 min, remove supernatant as possible with pipet, add 200 μl precooled Buffer A for every 20 μl compacted cell volume (add 1 μl DTT, 10 μl PMSF, and 1 μl protease inhibitor for every 1 mL Buffer A before use), The maximum rotational speed vortex vibrates violently for 15 s, and it is placed on ice for 10–15 min. Add 11 μl precooled Buffer B, swirl vigorously for 15 s at maximum rotating speed, and place on ice for 1 min. The solution was centrifuged at 4°C for 14,000 g for 5 min. At this time, it could be seen that the solution was divided into three layers: The lowest transparent layer, on which was white nucleus precipitate, and then the supernatant. The supernatant is transferred to another clean micro-centrifuge tube precooled as soon as possible and placed on ice to obtain cytoplasmic protein. The supernatant is packed and stored at –80°C to avoid repeated freeze-thaw. Insert the tip of a SPAR into the bottom of the centrifuge tube, suck out the bottom liquid and discard it. Add 100 μl of precooled Buffer C to the centrifuge precipitates (add 1 μl of DTT, 10 μl of PMSF, and 1 μl of protease inhibitor for every 1 mL of Buffer C before use). The maximum rotating speed of the vortex vibrated violently for 10 s, and the vortex was placed in an ice bath in a shaker for 40 min, 150 times/min, and then vibrated again for 30 s. The supernatant was centrifuged at 4°C for 14,000 g for 5 min and transferred into a clean micro-centrifuge tube precooled as soon as possible to obtain nuclear protein. The supernatant was packed and stored at –80°C to avoid repeated freeze-thaw. At last, the nucleoplasmic distribution of *EBF3*-WT and *EBF3*-N197D was detected by Western blot (Mini-PROTEAN Tetra,^®^ Bio-Rad, United States).

Primary antibodies used were anti-Flag antibody (ab205606), anti-GAPDH antibody (ab8245), Anti-LaminA antibody (ab108595), Goat Anti-Rabbit IgG H&L (HRP) (ab6721), Rabbit Anti-Mouse IgG H&L (HRP) (ab6728), which were purchased from Abcam Company (Abcam, Cambridge, United Kingdom).

## Results

### The Clinical Phenotypes and Imaging Examination Results of the Patient

**FIGURE 1:**
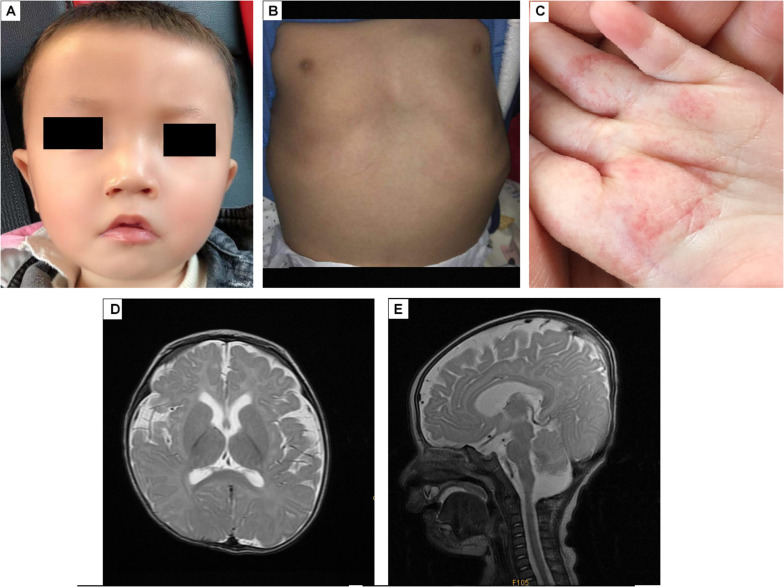
The appearance and MRI results of the patients. **(A)** The patient has an unclosed fontanelle, slight orbital hypertelorism, high nasal bridge, broad nasal tip, deep philtrum, and downturned mouth. **(B)** The patient has pectus excavatum. **(C)** The hemangiomas are in the palm of the left hand. **(D,E)** Brain MRI shows insignificant T2 hyperintensity at the bilateral anterior limbs of the internal capsule, enlargement of the bilateral lateral ventricles and lateral apertures.

The proband, a Chinese boy, was referred to the genetic counseling clinic of our hospital owing to global developmental delay. The patient was the first child and first birth, born through cesarean delivery at 39 weeks. All examinations during pregnance were normal. The proband’s birth length was 48 cm (3rd–10th centile); birth weight was 3.2 kg (25th–50th centile); head circumference was 34 cm (25th–50th centile); chest circumference was 32 cm; abdominal circumference was 29 cm. He presented with labored breathing, hypotonia, micropenis, microscrotum, palpable testis in the right inguinal canal, and impalpable left testis. Color ultrasound imaging of the patient’s bilateral scrotum, testes, and epididymis at birth elucidated an undescended testis in the left abdominal cavity, and a high mobility of the right testis with minor testicular hydrocele. Significant hypotonia was detected at 5 months. The patient could lift his head only at 10 months and underwent orchiopexy at 10 months. The first febrile seizure occurred at 1 year 2 months of age. The patient is now 1 year 6 months of age, his height is 78 cm (≤1 SD) and weight is 9.5 kg (≤1 SD). He was unsteady when sitting unassisted; could not climb, and could not say dad or mom; had an unclosed fontanelle; presented late teething; and exhibited slight orbital hypertelorism, high nasal bridge, broad nasal tip, deep philtrum, downturned mouth, and myopathic facies (stiff facial expressions) (Figure 1A). He also presented with pectus excavatum (Figure 1B), motor delay, intellectual disability, hemangiomas (in the palm of the left hand, Figure 1C), tracheomalacia, and feeding difficulties.

Brain MRI at 5 months (Figures 1D,E) revealed T2 hyperintensity in the bilateral anterior limbs of the internal capsule, suggesting potentially delayed myelination. Furthermore, enlargement of the bilateral lateral ventricles and lateral apertures, mega cisterna magna, bilateral mastoid, and middle ear effusions were observed. Brainstem auditory evoked potentials at 1 year 4 months of age showed prolonged latency of waves I, III, and V, and intervals and amplitudes were normal, suggested damage to the brain stem. No obvious abnormality was found in EEG.

Analysis of the proband’s karyotype from peripheral blood lymphocytes (400–500 kb) did not reveal any abnormality. The proband’s parents, who were non-consanguineous marriage, had normal clinical assessment, no exposure to toxic or harmful substances during pregnancy, and no family history of genetic diseases.

### Genome-Wide Copy Number Variation Analysis

No obvious microdeletion or microduplication was found.

### Identification of Candidate Mutations by Whole-Exome Sequencing

High-quality data were obtained through WES. The data of the proband, father, and mother were of 15.5, 14.4, and 15.8 Gb, the coverage of the target region was 99.78, 99.62, and 99.82%, and the average sequencing depth was 119, 131, and 144 reads, respectively. The percentage of the target region with an average sequencing depth of > 10 reads accounted for 98.02, 95.06, and 96.11%, respectively. After filtering variants according to the following criteria: (1) frequency < 1% according to the dbSNP, 1000 Genomes Project, ESP6500, and ExAC database; (2) protein-alteration or on canonical splice-sites; (3) homozygous, heterozygous, or *de novo* mutations; and (4) sequence variants interpreted in accordance with the guidelines of the American College of Medical Genetics and Genomics, and harmful variants were screened in accordance with the proband’s phenotype; an *EBF3* heterozygous mutation was detected in the proband but not in his parents.

### Validation via Sanger Sequencing

**FIGURE 2:**
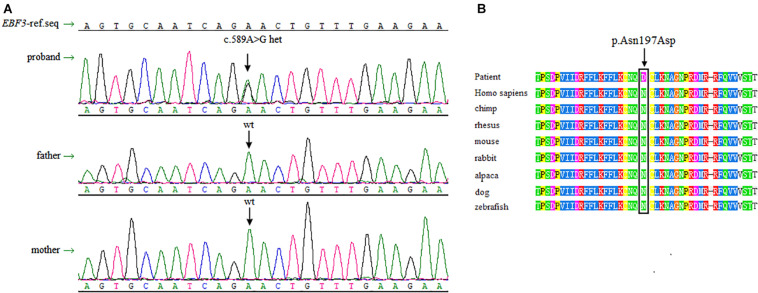
Sanger sequencing data and conservation of the amino acid residues around the mutation sites. **(A)** Sanger sequencing shows a heterozygous mutation of c.589A > G in *EBF3* is present in the proband, whereas both of the parents are wild-type for this variant. **(B)** The box shows the change is located at the conserved leucine residues. The mutated amino acid is highly conserved in 8 species.

Sanger sequencing was performed to validate this heterozygous mutation in the proband, and the mutation was not observed in the parents’ DNA isolated from peripheral blood lymphocytes (Figure 2A). The *EBF3* c.589A > G mutation was characterized by a substitution of Asn197 in CDS to Asp (p. Asn197Asp, p.N197D).

There is no record of this variant in the dbSNP, 1000 Genomes Project, ESP6500, and ExAC database. p.N197D is located in a highly conserved domain (Figure 2B). Multiple online software, including PolyPhen-2, SIFT, mutationtaster, and revel, predicted that this variant was pathogenic. Based on the ACMG guidelines for interpreting sequence variants, this variant was classified as a likely pathogenic variant. Specific evidences of pathogenicity for this variant includes: (1) PS2, *de novo* (both maternity and paternity confirmed) in a patient with the disease and no family history; (2) PM2, absent from controls (or at extremely low frequency if recessive) in Exome Sequencing Project, 1000 Genomes Project, or Exome Aggregation Consortium; (3) PP3, multiple lines of computational evidence support a deleterious effect on the gene or gene product (conservation, evolutionary, splicing impact, etc.).

### The Effects of the EBF3 Mutation at the Cellular Level

**FIGURE 3:**
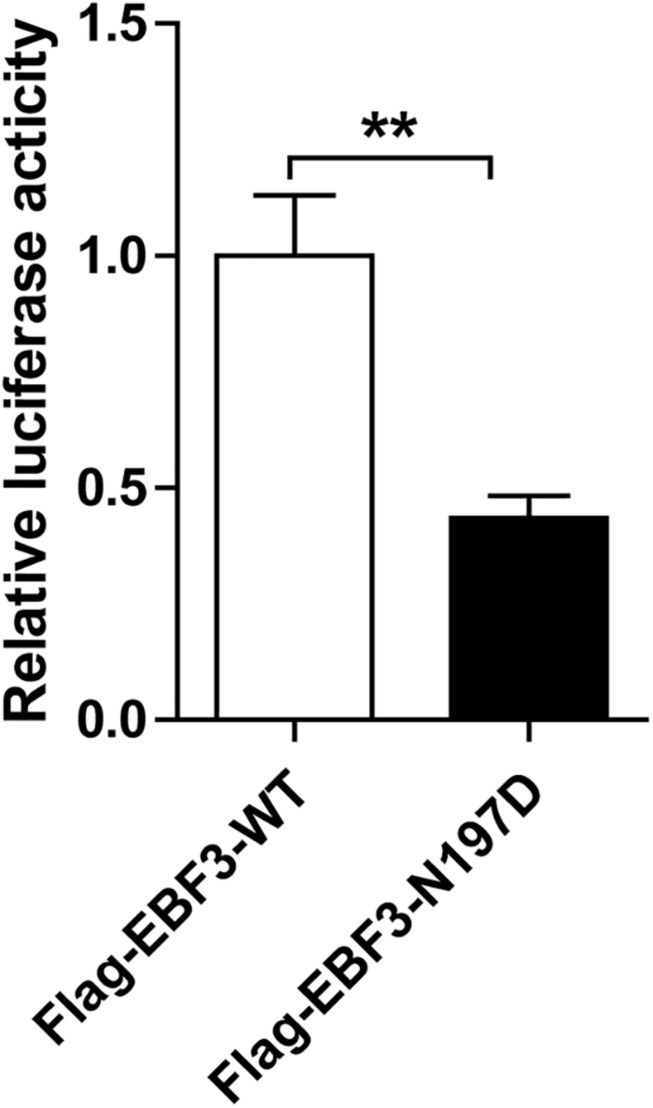
Transcriptional activity of EBF3-N197D mutant in transfected cells. The transcriptional activity of p21 promoter in EBF3-N197D mutant was decreased. Results were mean ± SD for three individual experiments which, for each condition, were performed in triplicate. **p < 0.01.

**FIGURE 4:**
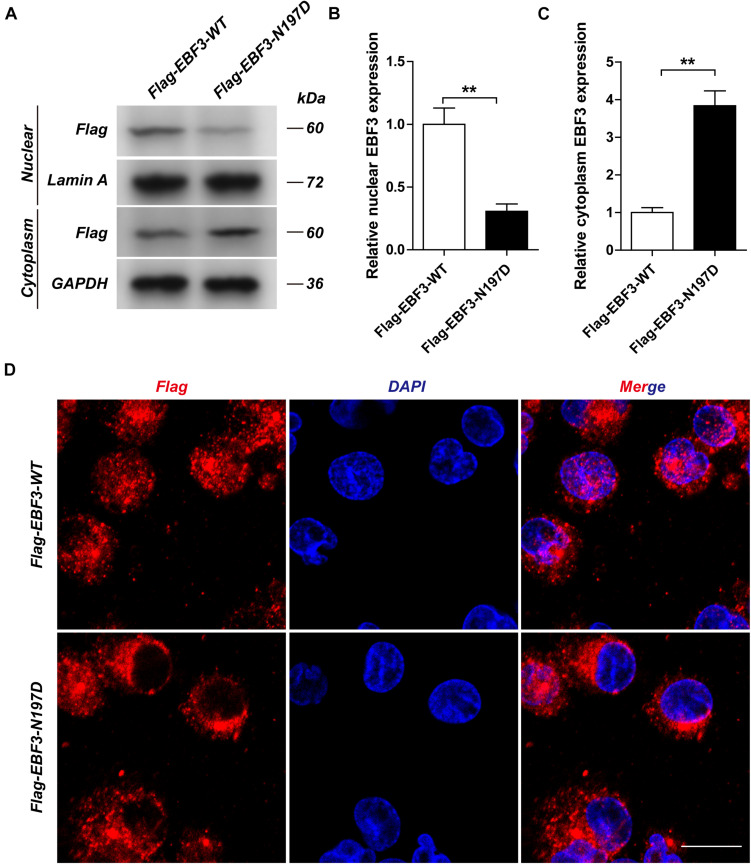
The localization of Flag-EBF3-WT and Flag-EBF3-N197D in HEK293T cells. The distribution of p.N197D mutant was significantly reduced in the nucleus **(A,B)**, and increased in the cytoplasm **(A,C)** and affected its entry into the nucleus **(D)**. Results were mean ± SD for three individual experiments which, for each condition, were performed in triplicate. **p < 0.01.

To test the transcriptional activity of p21 in *EBF3*-N197D, HEK293T cells were co-transfected with the p21 report plasmid and Flag-*EBF3*-WT or Flag-*EBF3*-N197D, and the luciferase signal was detected. Results were mean ± SD for three individual experiments which, for each condition, were performed in triplicate. In agreement with other studies (Jin et al., 2014; Harms et al., 2017), *EBF3*-N197D mutant showed impaired activation of luciferase reporter expression of the p21 promoter (Figure 3). In order to detect the effect of p.N197D mutant on protein localization, HEK293T cells were transfected with Flag-*EBF3*-WT or Flag-*EBF3*-N197D, and the localization of Flag-*EBF3*-WT and Flag-*EBF3*-N197D in HEK293T cells were detected by western blot assay and immunofluorescence assay. As shown in Figure 4, in contrast to the WT, the distribution of p.N197D mutant in nucleus was significantly reduced (*p* < 0.05, *p* < 0.01) (Figures 4A,B), and the mutant aggregated in the cytoplasm (Figures 4A,C) and affected its entry into the nucleus (Figure 4D).

## Discussion

**FIGURE 5:**
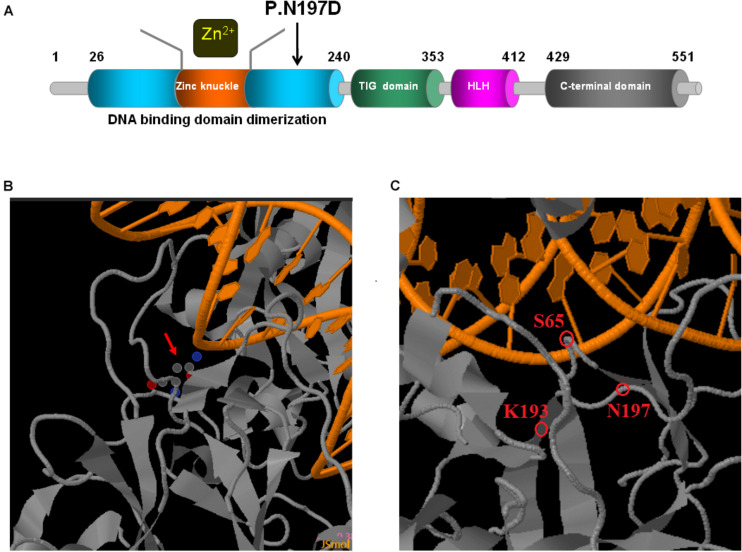
Schematic representations of EBF3 structure. **(A)** The EBF3 protein consists a DNA-binding domain with special zinc knuckle, an Ig-like/plexins/transcription factors domain, an atypical HLH domain, and a C-terminal transactivation domain. The mutant amino acid identified in this study is shown in black arrow. **(B,C)** Close up of DNA-binding interactions of the variant (generated with JSmol). The protein is shown in gray, and the DNA is shown in orange. Asn197 is interacted with DNA. Asn197 is close to Lys193 which participated in the electrostatic network of interactions with the DNA phosphate backbone and the carbonyl oxygen of Ser65.

*EBF3*, which encodes the early B-cell factor 3, is located in the q26 locus on chromosome 10 (Zardo et al., 2002). The protein is a highly conserved member of the Collier/Olf/EBF (COE) family of transcription factors, and is involved in neuronal differentiation, maturation, and migration. It is a necessary component in the development of the central nervous system (CNS) (Wang et al., 2004; Yamazaki et al., 2004). EBF3 contains a DNA-binding domain (DBD) with a unique zinc-finger-like conformation, an Ig-like/plexins/transcription factors (IPT/TIG) domain, an atypical helix-loop-helix (HLH) domain, and a C-terminal transactivation domain (TAD) (Figure 5A; Liberg et al., 2002; Siponen et al., 2010). Structural damage to the DBD potentially decreases the EBF1-like activity of EBF3 owing to haploinsufficiency (the DBD of EBF1 and EBF3 are highly homologous) (Feldhaus et al., 1992; Lukin et al., 2011). Furthermore, *EBF3* mutations potentially lead to the formation of abnormal EBF3-EBF2 heterodimers (EBF2 and EBF3 are highly homologous) (Sleven et al., 2017). *EBF3* p.Arg163Leu and p.Pro177Leu can partially damage the DBD, thus reducing its ability to bind to DNA, while also reducing the transcriptional activity of wild-type EBF3 through dominant negative effects (Lukin et al., 2011; Sleven et al., 2017). However, nonsense variants are predicted to undergo nonsense-mediated mRNA decay *in vivo* (Harms et al., 2017; Sleven et al., 2017). Therefore, *EBF3* mutations may exert their effects through loss of function or dominant negative regulatory mechanisms (Lukin et al., 2011; Chao et al., 2017; Sleven et al., 2017).

Upon validation via trio whole-exome sequencing and Sanger sequencing, we report a novel heterozygous missense mutation, c.589A > G, in *EBF3* in a boy with global developmental delay. JSMOL simulation of the EBF3 protein structure (Figure 5B) revealed Asn197 is located proximal to the DNA. Asn is a polar, uncharged amino acid, whereas Asp is a polar, negatively charged amino acid. Therefore, p.Asn197Asp can not only partially damage the DBD, but also affect the ability of EBF3 to recognize DNA, thereby decreasing its DNA-binding ability. Harms et al. (2017) reported that changes in p.Arg209Trp potentially alter Asn197 and reduce the binding affinity between Asn197 and DNA, thereby altering *EBF3*-mediated genetic regulation, inducing the downregulation of wild type *EBF3*, and eventually leading to a series of pathological conditions. The p.Asn197Asp mutation reported herein may directly affect the DNA-binding ability of this protein. In this study, the pathogenicity of the mutation was confirmed by cellular model. p.Lys193Asn, a variation closer to Asn197 (Figure 5C), reported by Sleven et al. (2017), could damage the electrostatic network of interactions with the DNA phosphate backbone and the carbonyl oxygen of Ser65, and activate Cd79a transcription. The p.Asn197Asp mutation might also disrupt the electrostatic network. Our study showed that *EBF3*-N197D mutant impaired activation of luciferase reporter expression of the p21 promoter (Figure 3) which was consistent with previous studies (Jin et al., 2014; Harms et al., 2017). Besides, the mutant was significantly reduced in nucleus while increased in the cytoplasm which affected its entry into the nucleus (Figure 4). These data suggest that the decreased affinity between ASN197 and DNA may lead to the reduced nuclear presence of EBF3, thus leading to the decreased expression of reporter genes.

Thus far, 33 mutations of *EBF3* have been reported worldwide, all of which are *de novo* mutations, including missense, frameshift, and nonsense mutations and splice variants (Chao et al., 2017; Harms et al., 2017; Sleven et al., 2017). Its functional heterozygous deletion is associated with developmental defects in the nervous system, eventually leading to hypotonia, ataxia, and delayed development syndrome (HADDS, OMIM #617330) (Chao et al., 2017; Harms et al., 2017; Sleven et al., 2017; Tanaka et al., 2017). HADDS follows an autosomal dominant inheritance. We compared the clinical phenotypes of patients with HADDS previously reported. The common phenotypes shared by these patients include motor delay, hypotonia, intellectual disability, and retardation in language acquisition. Besides the typical HADDS symptoms, our patient presented with an unclosed fontanelle, delayed teething, minimal facial expressions, high nasal bridge, broad nasal tip, deep philtrum, downturned mouth, feeding difficulties, cryptorchidism, micropenis, and pectus excavatum, consistent with the variable symptoms of HADDS, thus providing novel evidence for this syndrome. Furthermore, our patient did not present with ataxia, probably because he was too young for ataxia to be manifested. Although most previously reported patients with HADDS presented with ataxia, this symptom was observed only at a later stage (Lukin et al., 2011; Chao et al., 2017; Harms et al., 2017; Sleven et al., 2017; Tanaka et al., 2017). Moreover, Harms reported that ataxia was absent in a boy aged 1 year 11 months and a 25-year-old female (Harms et al., 2017). The present patient further presented several unusual symptoms including hemangioma, mild hearing abnormalities, and tracheomalacia, which have not been reported. Ebf3-deficient mice reportedly died of respiratory failure due to diaphragmatic relaxation dysfunction within 12 h postpartum (Jin et al., 2014). In the present patient, tracheomalacia may have been caused by hypotonia. However, it remains unclear whether tracheomalacia, and mild hearing abnormalities were truly associated with this condition.

## Conclusion

We found a novel heterozygous *EBF3* mutation with Trio-WES in a Chinese boy with HADDS. By constructing the plasmid and transfecting HEK293T cells, *EBF3*-N197D mutant showed impaired activation of luciferase reporter expression of the p21 promoter, and the mutant was significantly reduced in nucleus while increased in the cytoplasm which affected its entry into the nucleus. Thus, this mutation was most likely the pathogenic mutation for this individual. This syndrome is yet to be reported in Asia. To the best of our knowledge, this study is the first to report hemangiomas, tracheomalacia, and mild hearing abnormalities in HADDS caused by an *EBF3* mutation. However, only a few cases have been reported and further assessments of a larger number of patients are required to determine whether these symptoms are associated with HADDS. This study further validated the association between *EBF3* pathogenic mutations and HADDS. It provides key information regarding the pathogenic mutation spectrum of HADDS, which will facilitate the clinical diagnosis and genetic counseling of patients with HADDS. More case studies are needed to clarify genotype-phenotype interactions in HADDS.

## Data Availability Statement

The datasets presented in this study can be found in online repositories. The names of the repository/repositories and accession number(s) can be found in the article/supplementary material.

## Ethics Statement

This study fully complied with the tenets of the Declaration of Helsinki and has been approved by the Ethics Board of the Women’s and Children’s Hospital affiliated to Xiamen University, China. Written informed consent to participate in this study was provided by the participants’ legal guardian/next of kin. Written informed consent was obtained from the individual(s), and minor(s)’ legal guardian/next of kin, for the publication of any potentially identifiable images or data included in this article.

## Author Contributions

YH: conceptualization and writing—original draft. LM, YW, HY, XM, and YG: data curation. YH, HY, XM, JZ, MC, and LM: formal analysis. YH and LM: funding acquisition. YH and JZ: methodology. LM: supervision. YG and LM: project administration. YG, PL, and YZ: writing—review and editing. All authors read and approved the final manuscript.

## Conflict of Interest

The authors declare that the research was conducted in the absence of any commercial or financial relationships that could be construed as a potential conflict of interest.
